# Resource utilization and cost of influenza requiring hospitalization in Canadian adults: A study from the serious outcomes surveillance network of the Canadian Immunization Research Network

**DOI:** 10.1111/irv.12521

**Published:** 2018-01-24

**Authors:** Carita Ng, Lingyun Ye, Stephen G Noorduyn, Margaret Hux, Edward Thommes, Ron Goeree, Ardith Ambrose, Melissa K. Andrew, Todd Hatchette, Guy Boivin, William Bowie, May ElSherif, Karen Green, Jennie Johnstone, Kevin Katz, Jason Leblanc, Mark Loeb, Donna MacKinnon‐Cameron, Anne McCarthy, Janet McElhaney, Allison McGeer, Andre Poirier, Jeff Powis, David Richardson, Rohita Sharma, Makeda Semret, Stephanie Smith, Daniel Smyth, Grant Stiver, Sylvie Trottier, Louis Valiquette, Duncan Webster, Shelly A. McNeil, the Serious Outcomes Surveillance Network of the Canadian Immunization Research Network (CIRN) Investigators, The Toronto Invasive Bacterial Diseases Network (TIBDN) Investigators

**Affiliations:** ^1^ ICON Plc Vancouver BC Canada; ^2^ Canadian Center for Vaccinology IWK Health Centre and Nova Scotia Health Authority Dalhousie University Halifax NS Canada; ^3^ GSK Mississauga ON Canada; ^4^ Takeda (formerly GSK) Oakville ON Canada; ^5^ McMaster University Hamilton ON Canada; ^6^ Centre Hospitalier Universitaire de Québec Québec QC Canada; ^7^ University of British Columbia Vancouver BC Canada; ^8^ Mount Sinai Hospital Toronto ON Canada; ^9^ North York General Hospital Toronto ON Canada; ^10^ The Ottawa Hospital Ottawa Hospital Civic Campus Ottawa ON Canada; ^11^ Health Sciences North Research Institute Sudbury ON Canada; ^12^ Centre de santé et de service sociaux de Trois‐Rivières Trois Rivières QC Canada; ^13^ Michael Garron Hospital Toronto ON Canada; ^14^ Department of Infectious Diseases and Medical Microbiology William Osler Health System Brampton ON Canada; ^15^ McGill University Health Centre McGill University Montreal QC Canada; ^16^ University of Alberta Hospital Edmonton AB Canada; ^17^ The Moncton Hospital Moncton NB Canada; ^18^ Université de Sherbrooke Sherbrooke QC Canada; ^19^ Horizon Health Saint John NB Canada

**Keywords:** burden of illness, Canada, direct service costs, disease surveillance, hospital costs, human, influenza

## Abstract

**Background:**

Consideration of cost determinants is crucial to inform delivery of public vaccination programs.

**Objectives:**

to estimate the average total cost of laboratory‐confirmed influenza requiring hospitalization in Canadians prior to, during, and 30 days following discharge. To analyze effects of patient/disease characteristics, treatment, and regional differences in costs.

**Methods:**

Study utilized previously recorded clinical characteristics, resource use, and outcomes of laboratory‐confirmed influenza patients admitted to hospitals in the Serious Outcomes Surveillance (SOS), Canadian Immunization Research Network (CIRN), from 2010/11 to 2012/13. Unit costs including hospital overheads were linked to inpatient/outpatient resource utilization before and after admissions.

**Results:**

Dataset included 2943 adult admissions to 17 SOS Network hospitals and 24 Toronto Invasive Bacterial Disease Network hospitals. Mean age was 69.5 years. Average hospital stay was 10.8 days (95% CI: 10.3, 11.3), general ward stays were 9.4 days (95% CI: 9.0, 9.8), and ICU stays were 9.8 days (95% CI: 8.6, 11.1) for the 14% of patients admitted to the ICU. Average cost per case was $14 612 CAD (95% CI: $13 852, $15 372) including $133 (95% CI: $116, $150) for medical care prior to admission, $14 031 (95% CI: $13 295, $14 768) during initial hospital stay, $447 (95% CI: $271, $624) post‐discharge, including readmission within 30 days.

**Conclusion:**

The cost of laboratory‐confirmed influenza was higher than previous estimates, driven mostly by length of stay and analyzing only laboratory‐confirmed influenza cases. The true per‐patient cost of influenza‐related hospitalization has been underestimated, and prevention programs should be evaluated in this context.

## 1 INTRODUCTION

Influenza is a common, highly communicable disease associated with febrile upper and lower respiratory tract infection that can result in serious complications particularly in young children, pregnant women, the elderly, and those with underlying medical conditions.^1^ Influenza represents a substantial economic and clinical burden to the healthcare system, with a demonstrable relationship between the circulation of influenza virus and increased healthcare utilization.^2^

Severe cases of influenza requiring hospital admission represent the largest component of healthcare costs in the management of influenza,^2^ with up to 12 200 attributable hospitalizations annually in Canada.^3^ Estimated hospitalization costs vary substantially between Canadian provinces, possibly due to geographic differences in influenza strain circulation, standard practices for managing treatment, hospital unit costs, and methodologies used for allocating fixed costs.^4^, ^5^, ^6^ Previous studies of hospitalization across Canada for patients with a diagnosis of influenza‐like illness (ILI) have found lengths of stay and costs ranging from 3.7 days and costs of $2049 in Manitoba^6^ to 5.9 days in hospital and costs of $7664 in Ontario,^4^ and mean cost per case of $2145 across Canada as a whole.^4^, ^5^, ^6^

Understanding cost determinants and their geographic variation is a key to informing the delivery of public vaccination programs. The SOS Network has collected patient demographics and clinical characteristics, as well as treatment and health resource use prior to hospital admission, during hospital stay, and over 30 days following discharge for patients hospitalized with laboratory‐confirmed influenza. Data from the SOS Network demonstrated that the length of hospital stay (average 10.8 days) was substantially longer than that reported in prior Canadian studies of ILI diagnoses.^5^, ^6^ All regions of Canada are represented although the majority of cases were admitted in Ontario and Quebec.

The objective of this study was to estimate the average direct cost of hospitalization with laboratory‐confirmed influenza in Canadian adults by assigning unit costs to detailed resource utilization data collected prior to, during, and for 30 days after hospitalization in a cohort of adults with laboratory‐confirmed influenza admitted to participating hospitals of the SOS Network over 3 influenza seasons. Further objectives were to identify the influence of patient and disease characteristics, management, and outcomes on cost, and to explore variation in costs across geographic regions.

## 2 METHODS

The SOS Network conducted active surveillance for influenza among patients aged 16 years and over admitted to participating hospitals with acute respiratory illness. This dataset is comprised of patients with laboratory‐confirmed influenza admitted to the 17 participating SOS Network hospitals across 6 provinces and 24 associated sites of the Toronto Invasive Bacterial Disease Network (TIBDN) during the 2010/11, 2011/12, and 2012/13 influenza seasons. For each case, detailed demographic information, surgical history, medical comorbidities, details of hospital care, complications, and influenza outcomes were collected by interview and medical record review.^7^ In addition, disease characteristics such as influenza type and subtype were identified by reverse transcription polymerase chain reaction (RT‐PCR) on nasopharyngeal swab specimens.^7^

The SOS Network also collected data on resource utilization prior to hospital admission, during hospitalization, and for 30 days following discharge. Information was collected on any physician or emergency department visit prior to hospitalization. In order to tabulate in‐hospital resource use, general days‐on‐ward and intensive care unit (ICU) days‐on‐ward were calculated as the difference between admission and discharge dates. In all SOS Network sites, with the exception of 3 sites in Quebec, length of stay excluded days‐on‐ward designated as “alternate level of care” (ALC). Alternate level of care is a designation applied to on‐ward days spent once patients have been deemed ready for discharge, but who remain in hospital for factors unrelated to the reason for acute hospital care. In all 3 seasons, details of antiviral and antibiotic use prior to and during hospitalization and duration of mechanical ventilation, occurrence of complications, and ICU stay were collected. Following discharge, general days‐on‐ward and ICU days‐on‐ward for subsequent hospitalizations within 30 days were collected. Additional detailed information about the types and number of diagnostic tests and procedures performed in the hospital setting were only collected in the 2010/11 and 2011/12 seasons.^8^, ^9^

This study linked the resource use of patients with laboratory‐confirmed influenza enrolled by the SOS Network to a single set of unit price weights for each case, regardless of the hospital where treatment was received. These costs for hospital resource use were obtained from Hamilton Health Sciences (HHS), a conglomerate of 7 hospitals in Ontario. Hospital costs were received in the form of unit prices of hospital care incorporating department overheads and fixed costs. Fees for physician services were obtained from the Ontario Schedule of Benefits.^10^ Ward per‐diem costs were sourced from HHS and included costs for mechanical ventilation and supplemental oxygen; as well as, procedures conducted at the bedside such as intubation, but excluded laboratory tests, diagnostics, and imaging; unit costs for these were provided by HHS separately. Costs for antivirals and antibiotics used during hospitalization were based upon unit prices provided by the Queen Elizabeth II Health Sciences Centre formulary in Halifax, Nova Scotia.^11^ For pharmacy costs, expenditure related to the acquisition of medications was excluded and only components attributable to human resources and supplies were included. Costs of outpatient antiviral and antibiotic medications were based upon unit prices listed by the Ontario Drug Benefit (ODB) formulary.^12^ Dosing information was not collected by the SOS Network; consequently, the lowest recommended dose from product monographs for severe cases of lower respiratory tract infections was assumed.

### 2.1 Statistical methods

Surveillance data collected differed in detail for the included years. By design, the year with the most descriptive data was 2010/11, which included text descriptions of outpatient medications, laboratory tests, and diagnostic imaging and procedures. Subsequent years did not track this level of detail, but the surveillance datasets were designed to allow statistical extrapolation of these data. For seasons where cost of care components were not collected, multiple imputation was used to generate estimated components of cost based upon patients who were similar in age, stay in ICU, and presence of medical complications. The fully conditional specification (FCS) algorithm is a semi‐parametric imputation method that samples the multivariate model from a sequence of conditional regression models. FSC was used to create 5 imputation datasets that were combined to give final mean values for components.^13^

Patterns of resource use obtained from the SOS Network medical records review were combined with imputed data to provide a comprehensive picture of resource use. Costs were then linked to resource use patterns pre‐admission, during admission, and for 30 days post‐discharge. The total cost of an admission was calculated as the sum of the cost of ward and ICU stays, laboratory and other diagnostic tests, procedures, and medication costs. All costs are presented in 2015 Canadian dollars.

Means and 95% confidence intervals of hospitalization cost were reported for different geographic regions, subgroups, and overall. Costs between different subgroups were compared using *t* test and *P*‐values. To calculate an overall *P*‐value, an individual *P*‐value on each of the 5 imputed datasets was calculated first. The test statistics were then combined to generate an overall *P*‐value using Rubin's rule, the gold standard method to combine statistical tests using imputed data.^14^ The confidence intervals were estimated using a similar approach. Linear regression with backward selection was used to identify significant predictors of the total cost. Imputed and collected costs were combined with demographic and clinical characteristics, and influenza outcomes to explore determinants of influenza cost and the variation across Canadian treatment settings.

## 3 RESULTS

There were 2943 patients enrolled from 27 participating hospitals in 6 provinces over the included influenza seasons. The largest portion of the population was enrolled in Ontario (66.8%; 1966 patients) and Quebec (21.5%; 633 patients) with Eastern provinces (Nova Scotia and New Brunswick) contributing 197 patients (6.7%) and Western provinces (British Columbia, Alberta) contributing 147 patients (5.0%). The demographic and disease characteristics of the SOS Network study cohort show a relatively even male‐to‐female ratio (47.6%:52.4%) (see Table 1). Most patients had at least 1 comorbidity (90.3%), and nearly half the sample had chronic pulmonary illness (43.1%). Rates of past or current smoking were higher in the Eastern region than among the overall population enrolled (81.4% vs 50.5%). Patients hospitalized in the Western region were younger than the average age of the enrolled population (mean age 61.9 years vs 69.5 years).

**Table 1:** Patient and disease characteristics

	Western region	Ontario	Quebec	Eastern region	Full population
	N = 147 (5.0%)	N = 1966 (66.8%)	N = 633 (21.5%)	N = 197 (6.7%)	N = 2943 (100%)
Influenza Type					
Influenza A	117 (79.6%)	1530 (77.8%)	569 (89.9%)	176 (89.3%)	2392 (81.3%)
Influenza B	30 (20.4%)	436 (22.2%)	61 (9.6%)	21 (10.7%)	548 (18.6%)
Unknown Type	0 (0.0%)	0 (0.0%)	3 (0.5%)	0 (0.0%)	3 (0.1%)
Influenza A	117	1530	569	176	2392
Subtype H1	20 (17.1%)	177 (11.6%)	29 (5.1%)	12 (6.8%)	238 (9.9%)
Subtype H3	81 (69.2%)	1011 (66.1%)	292 (51.3%)	106 (60.2%)	1490 (62.3%)
Subtype Unknown	16 (13.7%)	342 (22.4%)	248 (43.6%)	58 (33.0%)	664 (27.8%)
Influenza B	30	436	61	21	548
Subtype VIC	6 (20.0%)	71 (16.3%)	14 (23.0%)	12 (57.1%)	103 (18.8%)
Subtype YAM	12 (40.0%)	257 (58.9%)	21 (34.4%)	0 (0.0%)	290 (52.9%)
Subtype Unknown	12 (40.0%)	108 (24.8)	26 (42.6%)	9 (42.9%)	155 (28.3%)
Female Gender	74 (50.3%)	1010 (51.4%)	347 (54.8%)	112 (56.9%)	1543 (52.4%)
Age (y)					
Mean (SD)	61.9 (19.7)	69.6 (19.2)	71.2 (18.1)	69.1 (17.3)	69.5 (19.0)
16‐49	42 (28.6%)	323 (16.4%)	88 (13.9%)	28 (14.2%)	481 (16.3%)
50‐64	37 (25.2%)	342 (17.4%)	97 (15.3%)	42 (21.3%)	518 (17.6%)
65‐75	23 (15.6%)	354 (18.0%)	114 (18.0%)	50 (25.4%)	541 (18.4%)
>75	45 (30.6%)	947 (48.2%)	334 (52.8%)	77 (39.1%)	1403 (47.7%)
Past or current smoker^a^	71 (52.6%)	861 (45.9%)	267 (55.6%)	153 (81.4%)	1352 (50.5%)
Obesity^a^	18 (18.2%)	353 (20.8%)	114 (25.2%)	62 (36.3%)	547 (22.6%)
Disease Characteristics					
Any comorbid condition	131 (89.1%)	1772 (90.1%)	563 (88.9%)	191 (97.0%)	2657 (90.3%)
Any pulmonary illness	61 (41.5%)	799 (40.6%)	284 (44.9%)	123 (62.4%)	1267 (43.1%)

N, number; SD, standard deviation.

a Percentages are calculated with unknown values removed. Smoking status unknown for 265 patients; obesity unknown for 527 patients.

The majority of patients (90.7%) were still alive 30 days post‐discharge and only 4.9% were re‐admitted to the hospital during follow‐up. Mean overall length of stay in hospital was 10.8 days (95% CI: 10.3, 11.3), comprising 9.4 days (95% CI: 9.0, 9.8) in a general ward (see Table 2). For the 14.4% of patients with an ICU stay, their average LOS in ICU was 9.8 days (95% CI: 8.6, 11.1). Total length of stay (LOS) and general ward LOS were consistent over the 3 influenza seasons considered. Mean total LOS was 12.6 days (95% CI: 11.2, 14.1) in 2010/11, 10.0 days (95% CI: 9.1, 10.9) in 2011/12, and 10.8 days (95% CI: 10.1, 11.4) in 2012/13. Mean general ward LOS was 9.9 days (95% CI: 8.7, 11.1) in 2010/11, 8.7 days (95% CI: 7.9, 9.5) in 2011/12, and 9.5 days (95% CI: 9.0, 10.0) in 2012/13.

**Table 2:** Treatment and outcomes

	Western region	Ontario	Quebec	Eastern region	Full population
	N = 147	N = 1966	N = 633	N = 197	N = 2943
Pre‐admission					
Physician visit (n, %)	60 (40.8%)	607 (31.2%)	120 (19.9%)	52 (26.4%)	839 (29.0%)
ED visit (n, %)	19 (12.9%)	227 (11.6%)	72 (11.4%)	26 (13.2%)	344 (11.7%)
During Hospital Stay					
Antibiotics on admission (n, %)	138 (93.9%)	1647 (83.8%)	536 (84.7%)	167 (84.8%)	2488 (84.5%)
General ward days (mean, SD)	9.0 (9.7)	8.6 (10.5)	11.5 (16.7)	10.8 (9.7)	9.4 (12.1)
ICU stay (n, %)	41 (27.9%)	264 (13.4%)	88 (13.9%)	31 (15.7%)	424 (14.4%)
ICU days if in ICU (mean, SD)	12.7 (15.4)	10.4 (14.5)	6.0 (5.3)	12.3 (11.7)	9.8 (13.1)
Mechanically ventilated (n, %)	30 (20.4%)	161 (8.2%)	49 (7.7%)	13 (6.6%)	253 (8.6%)
Following discharge					
Readmission days (mean, SD)	0.6 (3.2)	0.4 (2.5)	0.5 (3.0)	0.1 (1.0)	0.4 (2.6)
Outcomes					
Complications in hospital (n, %)	79 (53.7%)	1433 (73.0%)	179 (28.4%)	122 (61.9%)	1813 (61.7%)
30‐day readmission (n, %)	11 (13.1%)	101 (5.8%)	27 (5.2%)	5 (2.7%)	144 (5.7%)
Mortality (n, %)	16 (10.9%)	193 (9.8%)	48 (7.6%)	16 (8.1%)	273 (9.3%)

ED, emergency department; ICU, intensive care unit; n: number; SD: standard deviation.

For patients with an ICU stay, LOS in ICU declined over the course of 3 seasons; however, the decline was a trend, and not statistically significant. Mean ICU LOS was 14.3 days (95% CI: 10.3, 18.4) in 2010/11, 10.2 days (95% CI: 7.6, 9.4) in 2011/12, 8.8 days (95% CI: 7.3, 10.3) in 2012/13 (see Table S2). The overall cost of a case of laboratory‐confirmed influenza requiring hospitalization in Canada was estimated to be $14 612 (95% CI: $13 852, $15 372).

Across Canada, the cost of laboratory‐confirmed influenza requiring hospitalization was $13 711 (95% CI: $12 797, $14 625) in Ontario, $15 186 (95% CI: $13 705, $16 668) in Quebec, $17 132 (95% CI: $13 705, $16 668) in Eastern Canada, and $20 808 (95% CI: $15 798, $25 818) in Western Canada (see Table 3). The higher cost in Western Canada was largely driven by higher rates of ICU admission and longer ICU stays among those requiring ICU admission (see Table S2). Overall, a higher proportion of patients admitted in Western Canada required admission to ICU than in the other regions (27.9% in Western Canada vs, 13.4% in Ontario, 5.3% in Quebec, and 12.3% in Eastern Canada); mean ICU stay was also longer in Western Canada (12.7 days in Western Canada vs 10.4 days in Ontario, 6.0 days in Quebec, and 12.3 days in Eastern Canada (see Table 2, Table S2).

**Table 3:** Hospitalization costs in subgroups of interest

Patient group	N (%)	Overall LOS	Mean ($)	LCI ($)	UCI ($)
All	2943 (100)	10.8	14 612	13 852	15 372
Mortality					
Alive	2670 (90.7)	13.9	13 929	13 147	14 710
Dead	273 (9.3)	10.5	21 293	18 457	24 129
Stay in ICU					
Yes	424 (14.4)	19.8	39 477	35 664	43 289
No	2519 (85.6)	9.3	10 427	9990	10 863
Comorbidities					
Cardiac comorbidity	1216 (41.3)	11.9	15 206	14 193	16 218
Renal comorbidity	433 (14.7)	13.5	17 676	15 612	19 739
COPD	833 (28.3)	11.5	15 928	14 375	17 480
Regional subgroups					
Western	147 (5.0)	12.5	20 808	15 798	25 818
Ontario	1966 (66.8)	9.98	13 711	12 797	14 625
Quebec	633 (21.5)	12.4	15 186	13 705	16 668
Eastern	197 (6.7)	12.7	17 132	14 312	19 952

COPD, chronic obstructive pulmonary disease; ICU, intensive care unit; LCI, lower 95% confidence interval; LOS, length of stay; N, number; UCI, upper 95% confidence interval.

Univariate analysis found patients who experienced an ICU stay, renal comorbidity, or death had significantly higher hospitalization costs compared to the study average (see Table 3). The mean cost of influenza in patients admitted to the ICU was $39 477 (95% CI: $35 664, $43 289), compared to $10 427 (95% CI: $9990, $10 863) in patients admitted to general ward. The mean cost of treatment for patients who died within thirty days was $21 293 (95% CI: $18 457, $24 129) compared to a mean cost of patients who remained alive of $13 929 (95% CI: $13 147, $14 710). Sensitivity analyses were conducted to determine the effect of including mark‐ups and dispensing fees to outpatient medication and the exclusion of the Quebec sites that included ALC days in the LOS. When mark‐ups and dispensing fees were included, mean medication cost increased from $1.83 to $6.69 and overall costs increased from $14 612 to $14 617. When the 3 Quebec sites that included ALC in LOS were removed, overall costs decreased from $14 612 to $13 408, driven by the reduced cost of a ward stay.

## 4 DISCUSSION

The SOS Network dataset represents the most comprehensive national sentinel surveillance dataset available in Canada, providing prospectively collected data on health services utilization using standardized data collection tools across all SOS Network sites over 3 influenza seasons in a large cohort of adults admitted with laboratory‐confirmed influenza. The SOS Network provides the ideal dataset to explore the cost of laboratory‐confirmed influenza requiring hospitalizations across the country using a microcosting methodology.

The study reflects costs of laboratory‐confirmed influenza requiring hospitalizations and does not rely on non‐specific respiratory illness diagnoses obtained from administrative discharge data. By applying a single set of unit price weights to prospectively collected cases, we were able to compare cost of influenza hospitalization between regions in Canada and among several clinical risk groups.

Differences in the cost to treat a case of laboratory‐confirmed influenza requiring hospitalization were found across the Canadian provinces, and the source of this variation was explored. Hospitalization costs ranged from $13 711 in Ontario to $20 808 in Western Canada. Understanding the cost drivers associated with influenza hospitalization can inform policy and decision making regarding publicly funded influenza immunization programs.^5^, ^15^

The higher hospitalization costs in Western Canada were likely related to different practice patterns in that region. Patients treated in British Columbia and Alberta were more likely to be admitted to the ICU, which increased the cost per hospitalization. Hospitalization costs in Quebec were also higher than the national average, which may be related to differences in definition of ALC. Notably, some sites in Quebec included days in hospital following change in patient disposition to ALC as part of the original hospitalization. This practice would overestimate the LOS for these patients by including days where patients no longer required acute medical care, but who remained in hospital for other reasons, such as waiting for a bed in a long‐term care or assisted living facility. However, sensitivity analyses that excluded hospitals in which ALC days were included in the LOS resulted in a reduction in the average cost estimate from $14 612 to $13 408, suggesting that this was not a large contributor to the cost estimates.

The hospitalization cost estimated from the SOS Network data is higher than previously reported in national estimates by the Canadian Institute for Health Information (CIHI), and provincial estimates from Ontario^4^ and Manitoba.^6^ Manitoba and the CIHI reported estimates between $2049^6^ to $2415,^15^ respectively. The current study obtained unit costs with fully allocated overheads. When the costs were aggregated for all of the resource use in hospital, the average in‐hospital cost per day from the current analysis was $1254. Similarly, the Ontario Case Costing Initiative (OCCI) reported an average cost per day of $1338 for a cohort of patients admitted in 2011, which at the time of this current analysis was the most recent publicly available costs for patients admitted with influenza‐like illness to hospitals participating in the OCCI. The average cost for ILI admissions reported for OCCI hospitals was much lower at $7876 (inflated to 2015 $CAD) as the mean LOS was only 5.9 days (see Table S1).

The length of stay and cost shown for the SOS Network surveillance study cohort were greater than that reported in other Canadian sources of cost for ILI. The OCCI found across ILI diagnoses mean LOS ranging from 3.4 to 9.0 days and costs from $4090 to $13 000 was observed.^4^, ^16^ Costs provided through CIHI hospitals reporting across Canada use a case‐mix methodology, with weighting for resource intensity, based upon “most responsible diagnosis”, which has most contributed to the patient's stay in hospital.^5^ Costs in the Manitoba report were based upon the CIHI methodology, which may explain the similar values. The Manitoba case costing method also excludes physician services, ambulatory care, and hospital overheads such as administrative costs and capital costs of facilities.^6^ The costs from the Manitoba study include pediatric cases and show a trend toward increasing costs for ILI hospitalization as patients get older, similar to the OCCI. Neither the CIHI nor Manitoba report required that influenza be confirmed by laboratory testing.

The clearest driver of the higher hospitalization costs from the SOS Network data is LOS (see Table S1). While the overall cost and LOS from the SOS Network are larger than the estimates from OCCI, the cost per day calculated from the SOS Network ($1254) is lower than the cost estimated from the OCCI data ($1338)^4^.

Patients in the SOS Network study were hospitalized for an average of 10.8 days, compared with a mean stay of 5.9 days as reported by the OCCI^4^. While the LOS from the current study is longer than the previously published Canadian estimates, the LOS of 10.8 days is consistent with estimates from the United States, where studies have reported LOS of approximately 10 days.^17^, ^18^

Length of stay and costs derived by the SOS Network may be higher for several reasons, mostly related to the differences in measurement of diagnosis, age cohorts included, and national vs regional representation of cases. Notably, LOS fluctuates by ILI diagnosis, reflecting differences in disease severity and outcomes depending on the underlying etiology of ILI. Patients in the SOS Network study had laboratory‐confirmed influenza and are likely to be a more seriously ill population requiring longer mean LOS than the OCCI study which included patients with ILI without the requirement of laboratory confirmation of influenza. Differences in diagnosis classification may contribute to differences in LOS and cost. The CIHI report groups diagnoses by the case‐mix group (CMG+) methodology, under which influenza is included in a case‐mix under “influenza/acute upper respiratory infection.”^5^

The broad, active surveillance program implemented by hospitals involved in this study will result in a higher rate of influenza diagnoses than clinician‐directed testing, because admitted patients are routinely tested for influenza, regardless of the underlying condition that brought patients to the hospital. This study is likely to have a greater number of patients with underlying pulmonary comorbidities or pneumonia complications than previous studies because patients with apparent, explanatory diagnoses, such as COPD, are additionally tested for influenza. Patients with pulmonary comorbidities are more likely to have a longer LOS, one of the main cost drivers in our analysis. This may explain why this study found a higher LOS than in previously reported Canadian studies.

Finally, the SOS Network analysis accounted for resource utilization pre‐admission and during the 30‐day post‐discharge period, while estimates from Ontario, Manitoba, and CIHI only considered costs accrued during the primary hospitalization. However, costs associated with the pre‐admission and 30‐day post‐discharge periods were small compared to the cost of the primary hospitalization (Table 4).

**Table 4:** Overall costs per case of laboratory‐confirmed influenza requiring hospitalization

	Mean ($)	Lower 95% CI^a^ ($)	Upper 95% CI^a^ ($)
Pre‐hospital admission			
Physician visit	22	21	24
ED visit	109	—	—
Medications	1.83	1.43	2.22
Pre‐hospital admission costs	133	116	150
During hospital admission			
ED visit leading to admission	453	—	—
Hospital ward stay			
ICU stay	3772	—	—
General ward^b^	7720	—	—
Laboratory tests	409	358	460
Culture tests	700	656	743
VRE screens	685	627	742
MRSA screens	150	146	154
Diagnostic tests	11	10	11
Procedures	13	12	13
Medications	121	115	127
During admission costs	14 031	13 295	14 768
Readmission within 30 d			
Readmission hospitalization	322	246	397
Readmission ICU	126	—	—
Readmission costs	447	271	624
Total treatment cost	14 612	13 852	15 372

CI, confidence interval; ED, emergency department; ICU, intensive care unit; MRSA, methicillin‐resistant *staphylococcus aureus;* VRE, vancomycin‐resistant *enterococcus*.

a Costs with missing lower and upper 95% CI are based on resource use that includes imputed values.

b General ward stay includes general or intermediate care wards and includes days in original admitting hospital if transferred to the reporting hospital.

A strength of this study is the use of these fully allocated costs from a hospital corporate costing model for individual health resources within a hospital stay including laboratory cultures and viral screens, diagnostic tests, procedures and medications, as well as type of ward. The profile of health resource use during admission was combined with such unit prices, and health costs added for care prior to and after the index hospitalization to give a fully allocated cost for influenza case specific to each patient. A common source of unit costs was used because differences in hospital costing methodology would likely produce greater variation than the real variation in resource unit costs. The rich clinical data on patient characteristics, risk factors, and clinical outcomes for each case, along with use of fully allocated individual resource costs, allowed the variation in treatment patterns, clinical outcomes, and patient characteristics to be explored across Canada.

A further strength of this study is the validated imputation approach to impute missing cost components based on existing data, which allowed extrapolation of previous season's information to seasons which did not include this data. Exclusion of these components is equivalent to setting this cost to a value of zero, producing a bias toward underestimating the total cost.^19^, ^20^ A simpler, single‐imputation method that estimates 1 value and applies this estimated input to all cases with a particular missing data item used in other costing studies^19^ has been shown to artificially reduce variability.^21^, ^22^ A multiple imputation technique combines several datasets, with each incorporating estimates from existing cases that are similar in important prognostic factors, while retaining variation. The assumption that data are missing at random is upheld as changes in case reporting between seasons is the main reason for missing data.^23^ This statistical approach provides overall unbiased summary estimates and confidence intervals based on the correct estimate of variation.

There are several limitations to the datasets used within this study. The majority of the patients captured in the sentinel surveillance were treated in Ontario and Quebec, with no patients enrolled from Saskatchewan, Manitoba, Newfoundland and Labrador, or Prince Edward Island. Thus, the results from this study may not be reflective of the practice patterns and costs of hospitalization for laboratory‐confirmed influenza in these provinces. Further, the majority of patients were treated in university‐affiliated hospitals. Another limitation is the use of the Ontario Schedule of Benefits to inform fees for physician services, as physician fees vary from province to province. Additionally, the costs are derived from the unit prices provided from 1 hospital with a fully allocated costing system. It is unclear how costs would change if different unit prices were utilized. Further work could explore the application of microcosting data from a hospital outside Ontario, to confirm the applicability of findings across Canada. Lastly, our cost estimates may be conservative because we were not able to include costs associated with ALC days or attributable to continuing care (home supports and nursing home placement) that may have resulted from persistent declines in function following acute influenza illness, particularly in frail older adults. Future studies could measure the utilization of community‐based health resource utilization post‐hospitalization for laboratory‐confirmed influenza.

This study is the first to use resource use data and costs from Canadian adults with laboratory‐confirmed influenza collected during active outcomes surveillance. The overall cost per influenza hospitalization calculated was higher than previously published national estimates^5^ and provincial estimates from Ontario^4^ and Manitoba.^6^ The higher cost per hospitalization is largely driven by longer LOS than in previous estimates, rather than a higher cost per day. The cost per hospitalization calculated from this study reflects the cost of laboratory‐confirmed influenza cases. Previous estimates included all hospitalized cases with a clinical diagnosis, without requirement for laboratory confirmation, which likely contributed to overall shorter and less intensive hospitalizations and therefore lower costs. Influenza places a significant burden upon the healthcare system in Canada that has hitherto been underestimated.

## CONFLICT OF INTEREST

All authors participated in the design or implementation or analysis, interpretation of the study, and the development of this manuscript. All authors had full access to the data and gave final approval before submission. Carita Ng and Margaret Hux report payments to ICON plc from the GSK group of companies for the conduct of the study. Stephen Noorduyn and Edward Thommes report they were employed by the GSK group of companies during the conduct of the study. Melissa K Andrew reports grants from the GSK group of companies, Pfizer, and Sanofi, but no personal payments. Janet McElhaney reports payments from PCIRN for the conduct of the study, and payments to her institution from the GSK group of companies and Sanofi for her participation in advisory boards. Andre Poirier reports payments from Actelion, Sanofi‐Pasteur, and Genentech. Jeff Powis reports payments from the GSK group of companies, Merck, Roche, and Synthetic Biologics, outside the submitted work. Rohita Sharma is employed by the GSK group of companies and hold shares in the GSK group of companies. Louis Valiquette reports payments to her institution from the GSK group of companies for the conduct of the study. Grant Stiver reports receiving funding from the GSK group of companies for PCIRN research through the University of British Columbia. Todd Hatchette and Shelly A McNeil report payments to their institution from the GSK group of companies for the conduct of the study, and payments from Pfizer, Merck, Novartis, and Sanofi‐Pasteur, outside the submitted work. The following authors have nothing to disclose: May ElSherif, Lingyun Ye, Ron Goeree, Ardith Ambrose, Guy Boivin, William Bowie, Karen Green, Jennie Johnstone, Kevin Katz, Jason Leblanc, Mark Loeb, Donna MacKinnon‐Cameron, Anne McCarthy, Allison McGeer, David Richardson, Makeda Semret, Stephanie Smith, Daniel Smyth, Sylvie Trottier, and Duncan Webster.
