# Supplementary material for: Resource utilization and cost of influenza requiring hospitalization in Canadian adults: A study from the serious outcomes surveillance network of the Canadian Immunization Research Network
Source: Influenza Other Respir Viruses. 2018 Jan 24;12(2):232–40. doi: 10.1111/irv.12521 (PMC5820421; doi:10.1111/irv.12521)
Supplement: Supplementary file 1 [file IRV-12-232-s001.docx]

*Table S-I. Comparison of Hospitalization Costs: OCCI vs. SOS Network*

|  | **Ontario Case Costing Initiative**** | | | | | **Serious Outcomes Surveillance** | | | | |
| --- | --- | --- | --- | --- | --- | --- | --- | --- | --- | --- |
|  | **Cost ($)** | | | **LOS** | **Cost/**  **Day ($)** | **Cost ($)** | | | **LOS** | **Cost/**  **Day ($)** |
| **Age group** | **Mean** | **LCI** | **UCI** |  |  | **Mean** | **LCI** | **UCI** |  |  |
| 0 to 17 | 6,604 | 4,960 | 8,248 | 3.5 | 1,887 | N/A | N/A | N/A | N/A | N/A |
| 18 to 69 | 7,771 | 4,257 | 11,285 | 5.4 | 1,439 | 13,626* | 12,320 | 14,932 | 9.1 | 1,497 |
| 70 plus | 8,900 | 7,483 | 10,317 | 8.0 | 1,113 | 13,481 | 12,658 | 14,305 | 12.1 | 1,114 |
| All | 7,876 | 6,656 | 9,096 | 5.9 | 1,338 | 13,542 | 12,815 | 14,269 | 10.8 | 1,254 |

Note: *Results from the Serious Outcomes Surveillance Network (SOS Network) study have collapsed the 0 to 17 and 18 to 69 age groups due to a lack of patients in the 0 to 17 age group. The SOS Network group only enrolled patients 16 and over. **Cost estimates from the Ontario Case Costing Initiative have been inflated to $2015. The SOS Network costs presented do not include physician fees since OCCI cost estimates do not include physician fees.

**Abbreviations:** LCI: Lower Confidence Interval; LOS: length of stay; UCI: Upper Confidence Interval.

**Sources:** OCCI 2011,[4] Statistics Canada 2015.[16]
