# Supplementary material for: Resource utilization and cost of influenza requiring hospitalization in Canadian adults: A study from the serious outcomes surveillance network of the Canadian Immunization Research Network
Source: Influenza Other Respir Viruses. 2018 Jan 24;12(2):232–40. doi: 10.1111/irv.12521 (PMC5820421; doi:10.1111/irv.12521)
Supplement: Supplementary file 2 [file IRV-12-232-s002.docx]

*Table S-II – Length of stay by Region and Season*

|  | **Regions** | | | | | **Seasons** | | | |
| --- | --- | --- | --- | --- | --- | --- | --- | --- | --- |
|  | **Eastern**  **N = 197** | **Western**  **N = 147** | **Ontario**  **N = 1966** | **Quebec**  **N = 633** | **Total**  **N = 2,943** | **2010/11  N = 318** | **2011/12 N = 592** | **2012/13 N = 2,033** | **Total**  **N = 2,943** |
| **Total Length of Stay** |  |  |  |  |  |  |  |  |  |
| Mean | 12.7 | 12.5 | 10.0 | 12.4 | 10.8 | 12.6 | 10.0 | 10.8 | 10.8 |
| SD | 11.1 | 14.6 | 12.9 | 17.0 | 13.9 | 13.3 | 11.2 | 14.7 | 13.9 |
| Q25 | 6 | 4 | 4 | 5 | 4 | 5 | 4 | 4 | 4 |
| Median | 9 | 7 | 6 | 7 | 7 | 8 | 6 | 6 | 7 |
| Q75 | 15 | 14 | 11 | 13 | 12 | 14 | 11 | 11 | 12 |
| **General Ward Stay** |  |  |  |  |  |  |  |  |  |
| Mean | 10.8 | 9.0 | 8.6 | 11.5 | 9.4 | 9.9 | 8.7 | 9.5 | 9.4 |
| SD | 9.7 | 9.7 | 10.5 | 16.6 | 12.1 | 10.9 | 9.7 | 12.8 | 12.1 |
| Q25 | 5 | 3 | 3 | 4 | 4 | 4 | 4 | 4 | 4 |
| Median | 8 | 6 | 6 | 7 | 6 | 7 | 6 | 6 | 6 |
| Q75 | 13 | 11 | 9 | 12 | 10 | 11 | 9.5 | 10 | 10 |
| **ICU Admission** |  |  |  |  |  |  |  |  |  |
| Not admitted to ICU | 166 (84.3%) | 106 (72.1%) | 1702 (86.6%) | 545 (86.1%) | 2519 (85.6%) | 258 (81.1%) | 517 (87.3%) | 1,744 (85.8%) | 2519 (85.6%) |
| Admitted to ICU | 31 (15.7%) | 41 (27.9%) | 264 (13.4%) | 88 (13.9%) | 424 (14.4%) | 60 (18.9%) | 75 (12.7%) | 289 (14.2%) | 424 (14.4%) |
| **Length of ICU stay, if admitted to ICU** |  |  |  |  |  |  |  |  |  |
| Mean | 12.3 | 12.7 | 10.4 | 6.0 | 9.8 | 14.4 | 10.2 | 8.8 | 9.8 |
| SD | 11.7 | 15.4 | 14.5 | 5.3 | 13.1 | 15.6 | 11.4 | 12.8 | 13.1 |
| Q25 | 4 | 4 | 3 | 2 | 3 | 4 | 4 | 3 | 3 |
| Median | 7 | 8 | 6 | 4 | 6 | 10 | 7 | 5 | 6 |
| Q75 | 17 | 17 | 11 | 8 | 11 | 17 | 11 | 10 | 11 |

**Abbreviations:** ICU: intensive care unit; N: number; Q25: lower quartile, Q75: upper quartile, sd: standard deviation
